# Comparative analysis of different mobility aids in the early accelerated rehabilitation phase following acute achilles tendon repair surgery: a prospective cohort study

**DOI:** 10.1186/s13018-026-06881-6

**Published:** 2026-04-27

**Authors:** Zhuoqi Wei, Zengzhen Cui, Yuan Cao, Xiuzhi Li, Yuliang Fu, Liangyu Bai, Yang Lv

**Affiliations:** 1 Department of Orthopedics, Peking University Third Hospital, Haidian Qu, Beijing, 100191 China; 2 Engineering Research Center of Bone and Joint Precision Medicine, Haidian Qu, Beijing, 100191 China

**Keywords:** Acute Achilles tendon rupture, Surgical repair, Mobility aids, Accelerated rehabilitation

## Abstract

**Background:**

The objective of this study was to evaluate and compare the safety, efficacy, and cost-effectiveness of different mobility aids during the early rehabilitation phase following surgical repair of acute Achilles tendon rupture (AATR).

**Methods:**

This prospective cohort study included 198 patients who underwent surgical repair for AATR between April 2023 and February 2025, with 171 males (86.4%) and a mean age of 36.4 years. Based on the mobility aid used weeks 3–6 postoperatively, participants were categorized into four groups: Wheelchair (*n* = 43), Knee Scooter (*n* = 41), Axillary Crutches (*n* = 78), and Leg Support (LS) Walker (*n* = 36). Patients were scheduled for assessments at 2, 4, 6, 12, and 24 weeks, with an additional telephone follow-up conducted approximately one year after surgery. Primary outcomes included the rate of unplanned Emergency Department (ED) visits, the affected-to-unaffected (A: U) calf circumference ratio, and expected rehabilitation costs. Secondary outcomes encompassed the Visual Analog Scale, the Achilles Tendon Total Rupture Score, the American Orthopedic Foot & Ankle Society Ankle-Hindfoot Score, and time to key recovery milestones, including single-leg heel raise to 50% of contralateral side, return to light exercise, return to work, and return to pre-injury exercise. Continuous variables were analyzed using one-way ANOVA or Kruskal-Wallis test, and categorical variables using chi-square or Fisher’s exact test, while linear mixed-effects models were employed for longitudinal outcomes.

**Results:**

During the early rehabilitation phase (Weeks 3–6), unplanned ED visit rates differed significantly among the four groups (*p* = 0.032), with the Axillary Crutches showing significantly higher odds than the Wheelchair (OR = 8.24, 95% CI 1.16–58.53, *p* = 0.040). A significant difference in the A: U ratio was observed among the four groups at 6 weeks postoperatively (*p* = 0.037), and post-hoc comparisons showed significant differences for LS Walker vs. Wheelchair (*p* < 0.001), Axillary Crutches vs. Wheelchair (*p* = 0.002), and LS Walker vs. Knee Scooter (*p* = 0.038). The LS Walker and Axillary Crutches also demonstrated superior performance in VAS, ATRS, and AOFAS scores from Week 4 to Week 12, and achieved key recovery milestones significantly earlier (*p* < 0.001). Economic analysis revealed the lower direct treatment costs in the Axillary Crutches (477 RMB) and Knee Scooter (539 RMB), followed by the Wheelchair (664 RMB) and LS Walker (1126 RMB) (*p* < 0.001).

**Conclusions:**

LS Walker demonstrated superior efficacy in preserving muscle and accelerating functional recovery, while Knee Scooter offered a favorable profile for fall prevention and cost-effectiveness.

**Trial registration:**

ClinicalTrials.gov (NCT04663542), registered on 22 September 2020.

**Supplementary Information:**

The online version contains supplementary material available at 10.1186/s13018-026-06881-6.

## Background

Acute Achilles tendon rupture (AATR) is a common musculoskeletal injury, predominantly affecting physically active adults, with an annual incidence ranging from 10 to 35 per 100,000 individuals [1, 2]. The injury frequently occurs in Asian males aged 35 to 44 years and is associated with substantial impairment in walking ability, work participation, and sports performance [3, 4]. Given the pivotal role of the Achilles tendon in lower limb biomechanics, optimizing treatment and rehabilitation strategies is essential to restore function and minimize long-term disability [5–7].

Although controversy remains regarding operative versus nonoperative management of AATR, accumulating evidence suggests that surgical repair is associated with lower re-rupture rates and more predictable functional recovery, albeit at the cost of wound-related complications and postoperative tendon thickening on imaging [8–10]. The increasing adoption and ongoing refinement of minimally invasive surgical techniques have partially mitigated these concerns and shifted clinical focus toward postoperative rehabilitation [11–13]. In parallel, enhanced recovery after surgery (ERAS) protocols have emphasized early mobilization and progressive weight-bearing as key components of modern postoperative care [14, 15]. Early accelerated rehabilitation has been shown to improve regional perfusion, reduce muscle atrophy, and facilitate functional recovery when appropriately implemented [16].

Despite these advantages, early mobilization is not without risk. Premature return to sports or inadequately supported ambulation may increase the likelihood of falls, re-injury, and unplanned Emergency Department (ED) visits during the early postoperative period [17]. In this context, mobility aids play a central role in enabling ambulation while protecting the repaired tendon [18]. However, the choice of mobility aid varies widely in clinical practice, and existing literature has largely focused on rehabilitation protocols rather than the assistive devices used to implement them [19]. Evidence comparing different mobility aids with respect to safety, functional recovery, and healthcare costs during early rehabilitation after AATR repair remains limited.

Therefore, the selection of an appropriate mobility aid represents a clinically important yet underappreciated aspect of postoperative management. This prospective cohort study aimed to compare four commonly used mobility aids, including Wheelchair, Knee Scooter, Axillary Crutches, and Leg Support (LS) Walker, during the early rehabilitation phase following surgical repair of AATR. By evaluating safety outcomes, functional recovery, and direct medical costs, this study seeks to provide evidence-based guidance for optimizing mobility aid selection in clinical practice. We hypothesized that load-bearing mobility aids (LS Walker and Axillary Crutches) would be associated with superior functional outcomes and better calf muscle preservation compared to low-load aids (Wheelchair and Knee Scooter), but would also result in a higher incidence of unplanned ED visits due to falls during the early rehabilitation phase.

## Materials and methods

This study was approved by the Institutional Review Board of Peking University Third Hospital (IRB00006761-M2020315), and all participants provided written informed consent. The trial is registered at ClinicalTrials.gov (NCT04663542).

### Study design and participants

This observational prospective cohort study enrolled patients who presented to the ED of Peking University Third Hospital for AATR between April 2023 and February 2025. A total of 635 participants were assessed for eligibility, and 198 were ultimately included in this cohort study. Based on the mobility aid used in weeks 3–6 postoperatively, participants were categorized into four groups: Wheelchair (*n* = 43), Knee Scooter (*n* = 41), Axillary Crutches (*n* = 78), and LS Walker (*n* = 36). All participants completed the follow-up at 3 months, and 182 participants (92%) underwent the scheduled outpatient department assessment at 6 months after surgery.

**Fig. 1:**
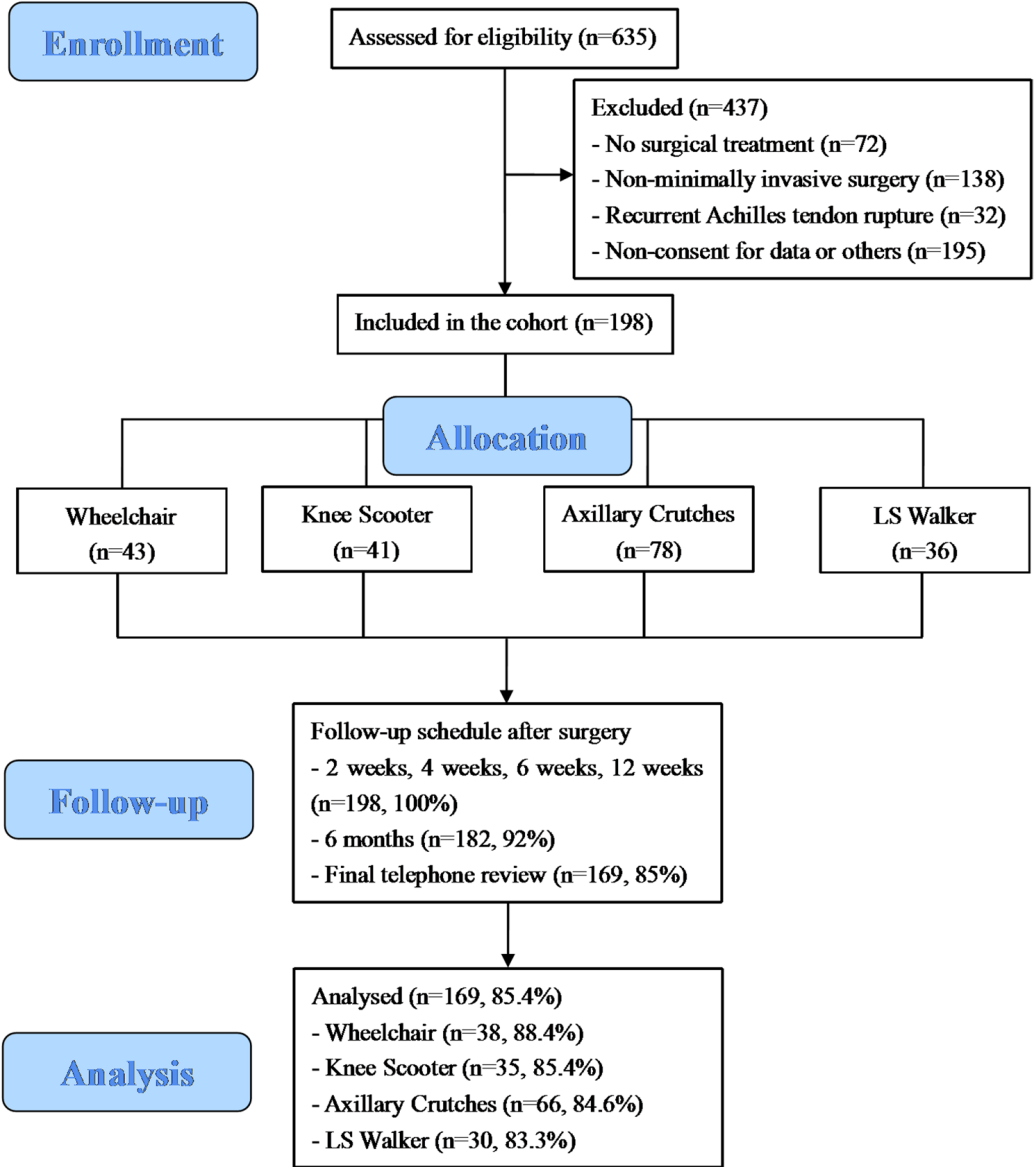
The flowchart of the study protocol. LS, leg support

Inclusion required: (1) age 18–60 years; (2) positive Thompson test with Magnetic Resonance Imaging or ultrasonographic confirmation of complete unilateral Achilles tendon rupture; (3) surgical management within 7 days of injury; (4) adherence to a standardized postoperative rehabilitation protocol; and (5) minimum 3-month follow-up completion. Exclusion criteria included: (1) diagnosed with ankle fractures or dislocation; (2) recurrent Achilles tendon rupture; (3) delayed presentation beyond 7 days post-injury; (4) pre-existing foot or ankle pathology, such as osteoarthritis; or (5) incomplete clinical data or follow-up duration of less than 3 months. The detailed flowchart of the study protocol is depicted in Fig. 1.

### Mobility aids

**Fig. 2:**
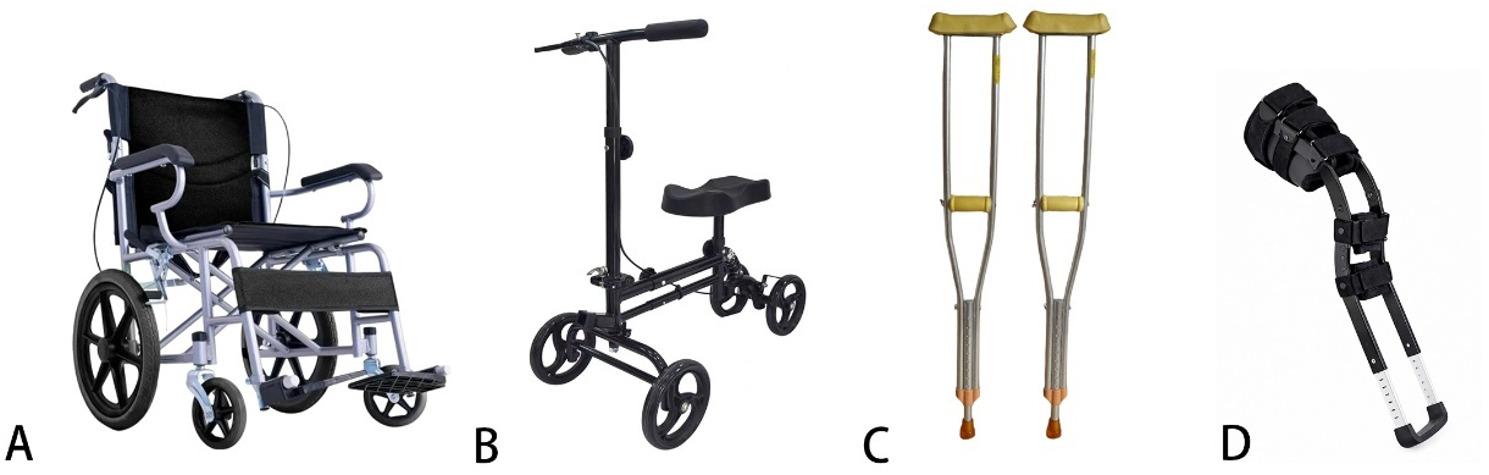
**A** Folding Mobility Wheelchair HY9920 (Cofoe, Changsha, Hunan, China) and **B** Knee Scooter MYDL-ZXQ (Maindl, Foshan, Guangdong, China) represent low-load aids. **C** Portable Axillary Crutches KFGZ034 (Cofoe, Changsha, Hunan, China) and **D** LS Walker ZX-II (Dabo Medical, Xiamen, Fujian, China) represent high-load aids. LS, leg support

Four mobility aids were utilized during the early rehabilitation phase (postoperative weeks 3–6) and categorized by load-bearing function: (1) Wheelchair (Folding Mobility Wheelchair HY9920, Cofoe, Changsha, Hunan, China), defined as a low-load aid ensuring non-weight-bearing ambulation; (2) Knee Scooter (Knee Scooter MYDL-ZXQ, Maindl, Foshan, Guangdong, China), a low-load aid transferring weight to the proximal tibia; (3) Axillary Crutches (Portable Axillary Crutches KFGZ034, Cofoe, Changsha, Hunan, China), enabling adjustable partial weight-bearing through upper limb support; and (4) LS Walker (LS Walker ZX-II, Dabo Medical, Xiamen, Fujian, China), a high-load aid designed for protected full weight-bearing through load transfer to the proximal lower limb (Fig. 2).

### Surgical procedures and Postoperative accelerated rehabilitation protocol

Under spinal anesthesia, patients were positioned prone with a tourniquet applied to the thigh. Prior to surgery, the Achilles tendon resting angle (ATRA) was measured bilaterally with the knee flexed at 90°, defined as the angle between the longitudinal axis of the fibula and the line connecting the fibular head to the fifth metatarsal head, representing an indirect indicator for assessing recovery and potential tendon elongation after repair [20, 21]. The primary surgical strategy for AATR was minimally invasive repair, unless specific contraindications warranted a modified open technique. The complete surgical protocols for both procedures have been explicitly documented in earlier literature [11, 22]. Following incision closure, all patients were immobilized in a below-knee brace equipped with a wedge to maintain the ankle in 30° of plantar flexion for two weeks, which was established in our previous research as the optimal duration for balancing functional recovery with the risk of postoperative complications [23]. All surgical procedures were performed by a single surgeon using a consistent operative technique. Upon complete removal of the brace, patients received brief on-site video training and personal demonstrations on mobility aid use in the outpatient clinic before being transitioned to the prescribed mobility aids with a controlled ankle motion (CAM) boot through week 6, and the early accelerated rehabilitation protocol was initiated, as detailed in Supplementary Table S1. In this study, the choice of mobility aid was determined through shared decision-making between the surgeon and the patient. Patients were informed of device-specific advantages, disadvantages, and costs, with final selection based on personal and practical factors.

### Data collection

Baseline demographic data, including sex, age, injured side, height, weight, and body mass index (BMI), along with injury-related information, were systematically recorded preoperatively. Patients were scheduled for outpatient assessments at 2, 4, 6, 12, and 24 weeks postoperatively. If an in-person visit was not feasible, a standardized telephone interview was conducted instead. A final follow-up was administered by telephone once patients reported resuming near pre-injury levels of physical activity. To ensure consistency, all evaluations were performed by the same attending foot and ankle trauma surgeon with 10 years of experience.

Primary outcome measures comprised the unplanned emergency department (ED) visits, the affected-to-unaffected calf circumference (A: U) ratio, and the expected direct medical costs among different groups. An ED visit was defined as an unscheduled presentation to the ED, commonly due to falls and related complications such as ankle sprain, re-rupture, or infection. The A: U ratio was used to quantify calf muscle atrophy, calculated by dividing the maximum circumference of the affected leg by that of the unaffected leg. All measurements were performed three consecutive times by the same investigator using a millimeter tape measure, and the average value was recorded. This relative measure obviates the limitations of absolute circumference, which is subject to high inter-individual variability. Then, the calculation of expected medical costs encompassed expenditures for mobility aids and those related to postoperative ED visits, including diagnostic imaging, medications, and additional treatments required. Secondary outcomes included patient-reported outcome measures (PROMs) and key recovery timelines. For PROMs assessment, the Visual Analog Scale (VAS), Achilles Tendon Total Rupture Score (ATRS), and American Orthopedic Foot & Ankle Society Ankle-Hindfoot (AOFAS) score were selected based on their established reliability and validity in evaluating functional recovery after AATR [24–26]. Recovery milestones were defined as follows: single-leg heel raise achieving 50% of the contralateral side (SLHR 50%), return to light exercise (RTLE), return to work (RTW), and return to pre-injury exercise (RTPIE). These milestones offer objective, performance-based criteria to monitor postoperative progress and have been widely used in previous studies [24, 27, 28].

Direct medical costs during the rehabilitation phase were estimated from a healthcare system perspective. Cost components included: (1) the acquisition cost of each mobility aid, based on institutional purchasing prices or market retail prices at the time of the study; (2) costs associated with unplanned ED visits, including consultation fees, diagnostic imaging, and medications, derived from hospital billing records; and (3) costs of managing complications, calculated by multiplying the frequency of each event by its corresponding unit cost based on institutional fee schedules or regional reimbursement standards. All costs are reported in Renminbi (RMB).

### Statistical analysis

All statistical analyses were performed using IBM SPSS Statistics (version 27.0), double-checked by two independent researchers, and any inconsistencies were resolved by consulting a third researcher to reach a consensus. Baseline characteristics were summarized as mean ± standard deviation (SD) or median (interquartile range, IQR) for continuous variables, and as frequencies with percentages for categorical variables. Normality of continuous data was assessed using the Kolmogorov-Smirnov test, and homogeneity of variances was evaluated using Levene’s test. For comparisons among the four groups, normally distributed variables were analyzed using one-way analysis of variance (ANOVA) followed by Tukey’s honestly significant difference (HSD) test for post-hoc pairwise comparisons. Non-normally distributed variables were compared using the Kruskal-Wallis H test, with Dunn’s test and Bonferroni correction applied for post-hoc analyses. Categorical variables were examined using the chi-square test or Fisher’s exact test, as appropriate. To account for within-patient correlations over time, linear mixed-effects models were applied to all longitudinal continuous outcomes, which included group, time, and their interaction as fixed effects, with random intercepts for each patient to capture individual variability. This approach utilizes all available observations and is robust to missing data under the missing-at-random assumption, allowing inclusion of all participants without imputation. Cross-sectional comparisons at specific time points were performed using one-way ANOVA or Kruskal-Wallis tests based on data normality. Adjusted analyses for binary outcomes were conducted using multivariable binary logistic regression models. A two-tailed p-value < 0.05 was considered statistically significant.

## Results

### Baseline characteristics of the participants

A total of 198 patients who received different mobility aids following surgical repair of AATR were enrolled in this study. The cohort included 171 males (86.4%), and the left side was affected in 103 individuals (52.0%). The mean age was 36.4 years, with individuals aged ≥ 50 years accounting for 10.6%. The mean height was 175.0 cm, the mean weight was 79.6 kg, and the mean BMI was 25.9 kg/m². There were 133 patients (67.2%) with a high pre-injury activity level and 35 patients (17.7%) with diabetes mellitus. No statistically significant differences were observed in these demographic characteristics among the four groups. Regarding injury mechanisms, 160 cases (80.8%) sustained AATR during athletic activities. The mean rupture gap distance (GDRS) was 1.9 cm, and the mean distance from the rupture site to the tendon insertion (DRSTI) was 4.3 cm. The average time to surgery was 3.2 days, with a mean operating time of 33.4 min and a hospital stay of 2.2 days. The clinical characteristics also showed no significant differences. The baseline demographic and clinical characteristics of the participants were summarized in Table 1.

**Table 1:** Baseline demographic and clinical characteristics of the participants

Variables	Wheelchair (*n* = 43)	Scooter (*n* = 41)	Crutches (*n* = 78)	Walker (*n* = 36)	*P* value
*Demographic*					
Age, years	36.8 ± 6.2	37.0 ± 6.1	35.9 ± 5.7	36.3 ± 6.1	0.182
< 50 years, n (%)	37 (86.0)	38 (92.7)	68 (87.2)	34 (94.4)	0.503
≥ 50 years, n (%)	6 (14.0)	3 (7.3)	10 (12.8)	2 (5.6)	
Male, n (%)	39 (90.7)	35 (85.4)	66 (84.6)	31 (86.1)	0.819
Left, n (%)	22 (51.2)	24 (58.6)	38 (48.7)	19 (52.8)	0.787
Height, cm	175.2 ± 5.1	174.9 ± 5.5	174.8 ± 4.7	176.5 ± 5.5	0.087
Weight, kg	78.8 ± 6.4	79.8 ± 5.9	79.5 ± 5.3	80.3 ± 5.9	0.104
BMI, kg/m^2^	25.7 ± 2.2	26.1 ± 2.1	25.9 ± 2.3	26.2 ± 2.1	0.238
*Activity level* ^a^ *, n (%)*					
Low (< 3 sessions/week)	19 (44.2)	13 (31.7)	25 (32.1)	8 (22.2)	0.234
High (≥ 3 sessions/week)	24 (55.8)	28 (68.3)	53 (67.9)	28 (77.8)	
Diabetes, n (%)	9 (20.9)	6 (14.6)	14 (18.0)	6 (16.7)	0.847
*Clinical*					
Sports injury, n (%)	36 (83.7)	33 (80.5)	61 (78.2)	30 (83.3)	0.191
GDRS, cm	1.9 ± 1.0	1.8 ± 1.2	1.9 ± 1.2	1.8 ± 1.1	0.432
DRSTI, cm	4.4 ± 0.9	4.2 ± 0.8	4.3 ± 0.8	4.4 ± 0.8	0.521
Time to Surgery, day	3.2 ± 1.2	3.1 ± 1.0	3.4 ± 1.2	3.2 ± 1.3	0.238
Operating Time, min	32.3 ± 3.3	34.5 ± 3.7	33.8 ± 3.3	33.5 ± 3.0	0.082
Length of Stay, day	2.0 ± 0.5	2.1 ± 0.5	2.2 ± 0.7	2.1 ± 0.5	0.158

Data are presented as mean ± SD or frequency (percentage). BMI, body mass index; GDRS, gap distance of the rupture site; DRSTI, distance from the rupture site to the Achilles tendon insertion. Activity level^a^ was dichotomized based on self-reported frequency of moderate-to-vigorous physical activity before injury, defined as activities substantially increasing heart rate or breathing, including jogging, ball sports, or heavy labor

### Unplanned emergency department visits

**Fig. 3:**
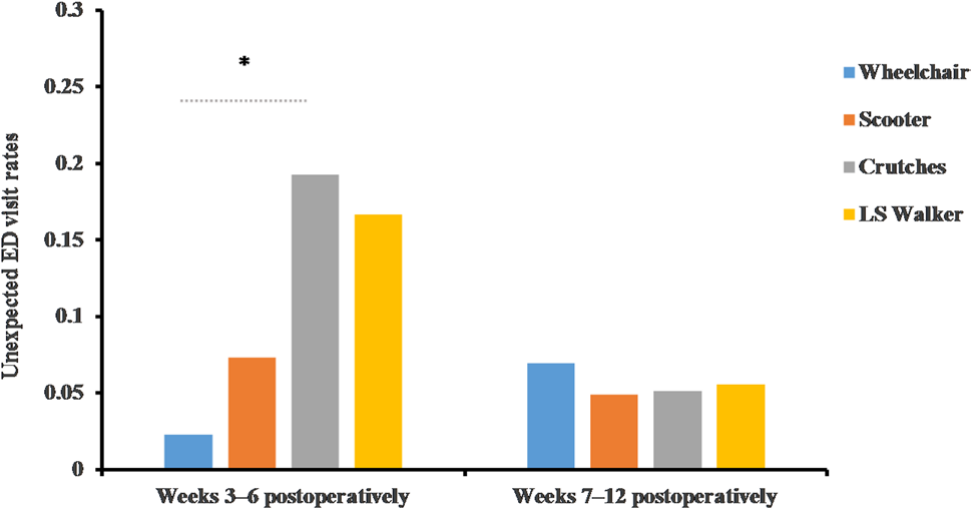
Unplanned ED visit rates among different mobility aids after AATR repair. During the period of Weeks 3–6, the rates of ED visits in descending order were as follows: Axillary Crutches (19.2%), LS Walker (16.7%), Knee Scooter (7.3%), and Wheelchair (2.3%). During the period Weeks 7–12, the four groups showed no significant difference in ED visit rates. ED, emergency department. LS, leg support. *, *p* < 0.05

During the early rehabilitation phase (weeks 3–6), unplanned ED visit rates differed significantly among the four groups (*p* = 0.032). Using the Wheelchair group as reference, the odds of an ED visit were significantly higher in the Axillary Crutches group (OR = 8.24, 95% CI 1.16–58.53, *p* = 0.040), whereas non-significant trends were observed in the LS Walker group (OR = 4.10, 95% CI 0.89–56.64, *p* = 0.085) and Knee Scooter group (OR = 3.13, 95% CI 0.38–25.82, *p* = 0.356). No significant intergroup differences were observed during the subsequent rehabilitation phase (weeks 7–12) (Fig. 3). The specific causes of unplanned ED visits within 12 weeks postoperatively are detailed in Table 2.

**Table 2:** Causes of unplanned ED visits within 12 weeks postoperatively

Consequences, *n* (%)	Wheelchair (*n* = 43)	Scooter (*n* = 41)	Crutches (*n* = 78)	Walker (*n* = 36)	*P* value
Without injury	1 (2.3)	2 (4.9)	6 (7.7)	5 (13.9)	0.224
Re-rupture	0 (0)	1 (2.4)	3 (3.8)	0 (0)	0.398
Ankle sprain	0 (0)	1 (2.4)	5 (6.4)	1 (2.8)	0.299
Wound infection	1 (2.3)	0 (0)	2 (2.6)	1 (2.8)	0.780
Delayed healing	1 (2.3)	1 (2.4)	2 (2.6)	1 (2.8)	0.995
Venous thrombosis	2 (4.7)	1 (2.4)	1 (1.3)	0 (0)	0.476
Total	5 (11.6)	6 (14.6)	19 (24.4)	8 (22.2)	0.297

Data are presented as frequencies and percentages. ED, emergency department

### A: U ratio of calf circumference

**Fig. 4:**
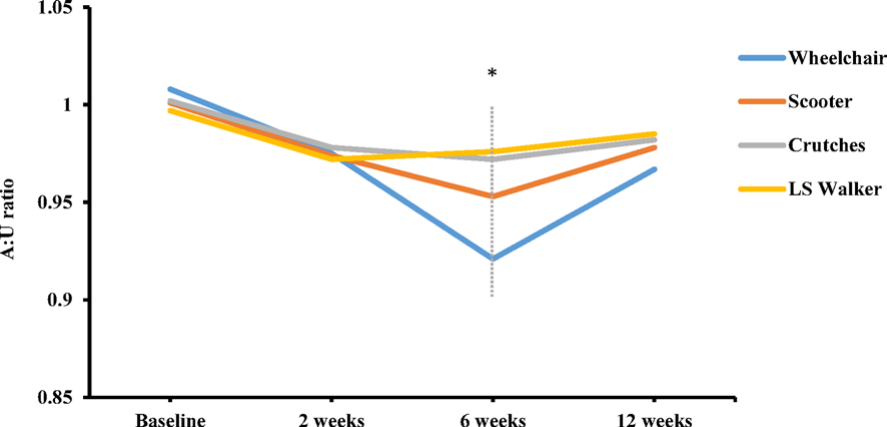
A: U ratio among different mobility aids after AATR repair. The A: U ratio showed no significant differences preoperatively or at 2 weeks postoperatively. At 6 weeks, the A: U ratio in descending order was as follows: LS Walker (97.8%), Axillary Crutches (97.2%), Knee Scooter (95.3%), and Wheelchair (92.1%). At 12 weeks postoperatively, no significant difference in the A: U ratio was detected among the groups. A: U ratio, the affected-to-unaffected calf circumference ratio. LS, leg support. *, *p* < 0.05

The A: U ratio was comparable across all groups at baseline (1.01 ± 0.05 vs. 1.00 ± 0.06 vs. 1.01 ± 0.04 vs. 0.99 ± 0.05, *p* = 0.169) and at brace removal (0.98 ± 0.06 vs. 0.98 ± 0.04 vs. 0.98 ± 0.05 vs. 0.97 ± 0.05, *p* = 0.675). Following the commencement of the accelerated rehabilitation protocol, the LS Walker and Axillary Crutches groups exhibited significantly less muscle atrophy compared to the Knee Scooter and Wheelchair groups. This difference peaked at 6 weeks (0.98 ± 0.03 vs. 0.97 ± 0.05 vs. 0.95 ± 0.03 vs. 0.92 ± 0.04, *p* = 0.037), and post-hoc analysis showed significant differences for LS Walker vs. Wheelchair (*p* < 0.001), Axillary Crutches vs. Wheelchair (*p* = 0.002), and LS Walker vs. Knee Scooter (*p* = 0.038). No statistically significant intergroup differences were observed by the 3-month follow-up (Fig. 4).

### Patient-reported outcome measures

For VAS pain scores, statistically significant differences among the four groups (LS Walker, Axillary Crutches, Knee Scooter, and Wheelchair) were observed at 4 weeks (1.5 ± 0.6 vs. 1.5 ± 0.8 vs. 1.8 ± 0.6 vs. 2.0 ± 0.5, *p* < 0.001) and at 6 weeks (0.5 ± 0.6 vs. 0.4 ± 0.5 vs. 0.7 ± 0.6 vs. 0.8 ± 1.0, *p* < 0.001). At 12 weeks postoperatively, no significant differences were detected among the groups. Regarding ATRS, significant intergroup differences were found at 4 weeks (46.5 ± 5.2 vs. 47.2 ± 5.5 vs. 39.6 ± 5.3 vs. 36.8 ± 6.2, *p* < 0.001), 6 weeks (63.2 ± 5.8 vs. 62.4 ± 4.9 vs. 57.3 ± 5.8 vs. 48.3 ± 5.1, *p* < 0.001), 12 weeks (83.5 ± 6.1 vs. 81.7 ± 5.8 vs. 77.4 ± 5.7 vs. 73.2 ± 4.7, *p* < 0.001), and 24 weeks (92.4 ± 4.9 vs. 91.8 ± 5.0 vs. 89.2 ± 4.2 vs. 86.3 ± 4.3, *p* < 0.001). Similarly, the AOFAS scores revealed statistically significant intergroup differences at 4 weeks (84.5 ± 9.2 vs. 83.8 ± 10.2 vs. 80.1 ± 7.3 vs. 78.3 ± 8.5, *p* < 0.001), at 6 weeks (92.3 ± 7.6 vs. 90.1 ± 5.3 vs. 84.6 ± 7.9 vs. 82.5 ± 8.1, *p* < 0.001), and 12 weeks (98.3 ± 5.9 vs. 98.5 ± 4.1 vs. 95.3 ± 4.8 vs. 92.6 ± 7.4, *p* < 0.001) postoperatively (Table 3).

**Table 3:** Patient-reported outcome measures after achilles tendon repair surgery

Outcome measures	Wheelchair (*n* = 43)	Scooter (*n* = 41)	Crutches (*n* = 78)	Walker (*n* = 36)	*P* value
*VAS, scores*					
Baseline	6.3 ± 1.1	6.4 ± 1.2	6.2 ± 1.2	6.3 ± 0.9	0.524
At 2 weeks	2.8 ± 0.7	2.8 ± 1.0	2.7 ± 1.1	2.9 ± 0.8	0.127
At 4 weeks	2.0 ± 0.5	1.8 ± 0.6	1.5 ± 0.8	1.5 ± 0.6	< 0.001
At 6 weeks	0.8 ± 1.0	0.7 ± 0.6	0.4 ± 0.5	0.5 ± 0.6	< 0.001
At 12 weeks	0.1 ± 0.3	0.1 ± 0.2	0.1 ± 0.3	0.1 ± 0.2	0.314
*AOFAS, scores*					
Baseline	46.8 ± 5.3	45.9 ± 3.7	45.1 ± 4.9	47.2 ± 5.5	0.422
At 2 weeks	68.7 ± 7.2	69.5 ± 7.6	69.3 ± 8.3	70.2 ± 8.6	0.293
At 4 weeks	78.3 ± 8.5	80.1 ± 7.3	83.8 ± 10.2	84.5 ± 9.2	< 0.001
At 6 weeks	82.5 ± 8.1	84.6 ± 7.9	90.1 ± 5.3	92.3 ± 7.6	< 0.001
At 12 weeks	92.6 ± 7.4	95.3 ± 4.8	98.5 ± 4.1	98.3 ± 5.9	< 0.001
At 24 weeks	98.2 ± 3.2	98.7 ± 2.5	99.3 ± 2.6	99.1 ± 1.7	0.063
*ATRS, scores*					
Baseline	21.2 ± 3.8	21.7 ± 4.1	22.3 ± 4.8	20.8 ± 4.4	0.612
At 2 weeks	25.5 ± 3.9	25.8 ± 4.3	26.0 ± 5.1	25.1 ± 4.1	0.108
At 4 weeks	36.8 ± 6.2	39.6 ± 5.3	47.2 ± 5.5	46.5 ± 5.2	< 0.001
At 6 weeks	48.3 ± 5.1	57.3 ± 5.8	62.4 ± 4.9	63.2 ± 5.8	< 0.001
At 12 weeks	73.2 ± 4.7	77.4 ± 5.7	81.7 ± 5.8	83.5 ± 6.1	< 0.001
At 24 weeks	86.3 ± 4.3	90.2 ± 4.2	91.8 ± 5.0	92.4 ± 4.9	< 0.001

Data are presented as mean ± SD. VAS, Visual Analog Scale for pain. ATRS, achilles tendon total rupture score; AOFAS, American orthopedic foot & ankle society score hind-foot scale

### Recovery timeline

With respect to postoperative recovery timelines, the LS Walker and Axillary Crutches groups achieved recovery milestones earlier than the Knee Scooter and Wheelchair groups. This included significant differences in the time to SLHR 50% (10.3 ± 1.8 vs. 10.6 ± 1.9 vs. 12.3 ± 2.2 vs. 14.2 ± 2.3 weeks, *p* < 0.001), RTLE (14.5 ± 3.3 vs. 14.8 ± 3.6 vs. 16.2 ± 3.9 vs. 18.9 ± 4.1 weeks, *p* < 0.001), RTW (5.7 ± 1.0 vs. 5.8 ± 1.2 vs. 6.1 ± 1.4 vs. 6.2 ± 1.1 weeks, *p* = 0.048), and RTPIE (27.6 ± 5.8 vs. 28.3 ± 7.6 vs. 31.0 ± 5.4 vs. 32.5 ± 6.2 weeks, *p* < 0.001). The comparison of recovery timelines was detailed in Table 4.

**Table 4:** Recovery timeline

Recovery time, weeks	Wheelchair (*n* = 43)	Scooter (*n* = 41)	Crutches (*n* = 78)	Walker (*n* = 36)	*P* value
SLHR 50%	14.2 ± 2.3	13.3 ± 2.2	10.6 ± 1.9	10.3 ± 1.8	< 0.001
RTLE	18.9 ± 4.1	16.2 ± 3.9	14.8 ± 3.6	14.5 ± 3.3	< 0.001
RTW^a^	6.2 ± 1.1	6.1 ± 1.4	5.8 ± 1.2	5.7 ± 1.0	0.048
RTPIE^b^	32.5 ± 6.2	31.0 ± 5.4	28.3 ± 7.6	27.6 ± 5.8	< 0.001

Data are presented as mean ± SD. SLHR 50%, single-leg heel raise to 50% of the contralateral side. RTLE, return to light exercise. RTW^a^, return to work, but avoid excessive strenuous physical labor as much as possible. RTPIE^b^, return to pre-injury exercise intensity, obtained through an additional telephone follow-up

### Economic analysis

The expected total cost was highest in the LS Walker (1126 RMB), followed by the Wheelchair (664 RMB), Knee Scooter (539 RMB), and Axillary Crutches (477 RMB), based on summed expenditures for mobility aids, ED visits, and complications. Detailed cost breakdowns are shown in Table 5.

**Table 5:** Cost analysis of the rehabilitation phase

Categories	Price, RMB	Wheel chair	Scooter	Crutches	Walker	*P* value
Mobility aids		500	300	100	900	
ED visit	600	70	88	147	133	0.002
*Complications*						
Re-rupture	3000	0	74	116	0	< 0.001
Ankle sprain	500	0	13	33	14	< 0.001
Wound infection	800	19	0	21	23	< 0.001
Delayed healing	2000	47	49	52	56	0.034
Venous thrombosis	600	28	15	8	0	< 0.001
Sum^a^		664	539	477	1126	< 0.001

ED, Unplanned Emergency Department visits. Sum^a^, Estimated direct costs summary, calculated as: (Cost of Mobility Aid) + (Cost of ED Visit × Frequency) + ∑ (Unit Cost of Each Complication × Its Frequency)

## Discussion

This prospective cohort study provides a direct comparison of four mobility aids within an accelerated rehabilitation protocol following surgical repair of AATR. Our findings suggest that load‑bearing aids (LS Walker and Axillary Crutches) were associated with better muscle preservation and faster functional recovery, but also with a higher incidence of falls during the early rehabilitation phase. In contrast, low‑load aids (Wheelchair and Knee Scooter) offered improved safety at the expense of slower functional gains. Economic analysis further differentiated these options, with Axillary Crutches and Knee Scooter emerging as the most cost‑effective choices based on direct medical costs. These findings are primarily applicable to mobility aid selection during the early to mid-term recovery period.

Accidental falls represent a major clinical challenge during the early accelerated rehabilitation of AATR, especially when early weight-bearing and mobilization are permitted [18]. Falls are clinically significant because they are associated with tendon re-rupture, impaired wound healing, and surgical site complications. Limited research indicates that due to the restricted load-bearing capacity of the repaired Achilles tendon, the incidence of at least one accidental fall during the postoperative rehabilitation phase is estimated to range from 10% to 50% [29]. Traditionally, studies recommended six weeks of immobilization with restricted physical activity to protect the fragile repaired Achilles tendon [30]. Furthermore, Maffulli et al. recommended a more cautious, slowed-down rehabilitation protocol and demonstrated more favorable ATRA and ATRS outcomes at the 12-month follow-up [31]. However, the current paradigm is shifting towards early weight-bearing to expedite return to function, despite its association with an elevated risk of complications [32, 33].

Among fall-related complications, tendon re-rupture is frequently precipitated by sudden ankle dorsiflexion during stair- or height-related falls, and the intrinsically poor vascularity of the Achilles tendon predisposes it to impaired healing after re-injury [34]. In our investigation, patients utilizing Axillary Crutches or LS Walker demonstrated a higher incidence of postoperative falls following AATR, which may be attributed to suboptimal device configuration or insufficient training, a finding commonly observed in clinical practice and previously reported in the literature [35]. Besides, both mobility aids require substantial lower limb balance and coordination. Given that these capacities typically begin to decline around the fifth decade, age is likely to influence both the choice of aid and the effectiveness of its use [36]. Further stratified analysis by age revealed that the rate of unplanned ED visits was as high as 41.7% among older patients (≥ 50 years) using high-load aids (Supplementary Table S2). Interestingly, the LS Walker group experienced a higher incidence of uncomplicated falls without subsequent treatment than the Axillary Crutches group. This can be explained by the protective mechanism of the fixed position that can destabilize gait but simultaneously protect the repaired tendon from excessive tensile loads during a stumble [37, 38]. In fact, six patients in the LS Walker group experienced accidental falls between postoperative weeks 3 and 6. Of these, five patients underwent clinical evaluation and emergency radiological assessment, all of which confirmed the absence of secondary injuries; these falls were primarily attributed to unfamiliarity with the use of mobility aids and difficulties in maintaining balance. One patient sustained an ankle sprain, which was considered to be related to insufficient limb stabilization due to overly loose straps, resulting in slippage of the affected extremity. Additionally, in several cases from our cohort, wound infections occurred after falls. When antibiotic therapy is indicated, fluoroquinolones should be avoided, as these agents are associated with an increased risk of Achilles tendon disorders [39]. However, after discontinuation of mobility aids and recovery of neuromuscular control, intergroup differences in fall-related unplanned ED visit rates were no longer observed, indicating that the safety disadvantage of load-bearing aids is largely confined to the early postoperative period.

Calf muscle atrophy is a hallmark of AATR and a key determinant of functional recovery. While surgical repair reduces muscle wasting compared with nonoperative treatment, rehabilitation strategies and mechanical loading exposure critically influence the degree of muscle preservation [34, 40]. In this study, patients using leg support walkers or axillary crutches demonstrated significantly less calf muscle atrophy during the early postoperative period, as reflected by higher affected-to-unaffected calf circumference ratios. This difference is likely attributable to increased cumulative mechanical loading associated with weight-bearing mobility aids [41]. Beyond structured rehabilitation sessions, routine ambulation and daily activities performed with weight-bearing aids contribute to “incidental loading,” thereby promoting muscle activation and mitigating disuse atrophy. In contrast, mobility aids that restrict weight-bearing confine mechanical stimulation largely to supervised rehabilitation, reducing total daily loading exposure despite improved safety. The observed association between reduced muscle activity and a higher incidence of lower limb venous thrombosis among wheelchair users further supports the systemic consequences of prolonged unloading [42]. The existing literature established that venous thrombosis following AATR is linked to adverse long-term outcomes [43]. Muscle pump activation during weight-bearing ambulation may play a protective role in venous circulation, underscoring the broader physiological implications of mobility aid selection. This aligns with prior evidence showing that better calf symmetry, heel-rise function, and ankle motion are associated with more timely recovery of activity after Achilles repair [44].

Beyond statistical significance, the clinical relevance of observed differences in PROMs deserves consideration. The minimal clinically important difference (MCID) represents the smallest change that patients may perceive as meaningful [45]. For VAS pain scores, the intergroup differences observed at 4 weeks and 6 weeks appeared to fall below the established MCID range of 1.0–2.0 points previously reported for musculoskeletal conditions [46]. This may suggest that although statistically significant, these differences might not translate into clinically perceptible pain relief for patients. With respect to AOFAS scores, the magnitude of difference between load-bearing and low-load aids at 6 weeks exceeded the MCID threshold of 6–9 points [47], whereas the corresponding differences at 4 weeks and 12 weeks remained below this benchmark. Overall, clinically meaningful benefits were observed primarily for short-term functional improvements, whereas differences in pain and longer-term outcomes fell below the threshold likely to be perceived as beneficial by patients.

Lastly, another key consideration involves the direct costs during rehabilitation, which we calculated as the sum of ED visits, including the costs for magnetic resonance imaging and ultrasound scans, and complication management expenses. Westin et al. incorporated productivity loss as an essential component of indirect costs in the cost-effectiveness analysis following AATR [48]. While in our region, the time to RTW is concurrently determined by both Achilles tendon recovery and corporate sick leave policies, and thus, it was not included in our cost-effectiveness comparative analysis. Although evidence regarding the cost-effectiveness of different weight-bearing strategies after surgical repair of AATR remains limited, studies in non-operative populations have suggested that early, more aggressive weight-bearing and muscle activation may be associated with lower overall rehabilitation costs [26]. Axillary crutches are commonly favored because of their portability and low acquisition cost; however, their use has been associated with higher rates of injury-related complications, which may paradoxically increase total treatment expenditures [49]. In contrast, wheelchairs provide superior postoperative protection but are generally associated with slower functional recovery and less favorable functional outcomes. The LS walker has gained popularity in recent years as it facilitates earlier independent ambulation and has demonstrated favorable clinical performance; nevertheless, its relatively high cost limits its widespread adoption, and its use is more commonly observed among physically active or athletic patients.

Collectively, our findings suggest that mobility aid selection should be regarded as an integral component of postoperative rehabilitation planning rather than a purely logistical decision, and that this choice may also be influenced by factors such as age and pre-injury activity level. Load-bearing aids may be advantageous for patients prioritizing rapid functional recovery and muscle preservation, particularly younger and physically active individuals with adequate balance and supervision. Conversely, low-load aids may be more appropriate for patients at higher risk of falls or those emphasizing safety and cost containment during the early postoperative phase. Importantly, mobility aids should be viewed as dynamic tools, with the potential for staged transitions as patient capacity evolves. More importantly, appropriate instruction on the use of mobility aids and comprehensive patient education are essential. Future studies exploring adaptive or sequential mobility aid strategies may further optimize the balance between safety and recovery.

Several limitations should be acknowledged. First, the non-randomized allocation of mobility aids through shared decision-making and patient preference may have introduced selection bias and confounding. Patients with better baseline function or greater ambulation confidence may have been more likely to choose load-bearing aids, whereas those more concerned about instability or falls may have preferred more protective devices. Additionally, unmeasured factors such as rehabilitation adherence, home support, and risk awareness may have influenced both mobility aid selection and postoperative recovery. Although an additional adjusted analysis yielded broadly similar results (Supplementary Table S3), residual confounding cannot be fully excluded. Then, the disproportionate representation of male patients (86.4%) limits the applicability of our findings to female populations. Owing to limited availability of professional physical therapists, a standardized video-guided rehabilitation protocol was adopted, and its self-guided nature may have introduced variability in training intensity. Furthermore, only mobility aids commonly used in our region were evaluated, and regional practice patterns or combined use of multiple aids were not considered, potentially limiting generalizability. Meanwhile, the lack of systematic occupational data may have introduced confounding in RTW analyses, as sedentary workers typically return earlier than manual laborers irrespective of mobility aid used. Furthermore, limb loading during ambulation was not objectively quantified, and future studies using wearable sensors or gait analysis may provide more precise biomechanical insights. Finally, longer-term follow-up is required to determine whether early rehabilitation differences influence reinjury rates or long-term athletic performance.

## Conclusion

Mobility aid selection during early rehabilitation after acute Achilles tendon repair was associated with differences in safety and functional recovery. Load-bearing aids promoted earlier functional gains, while low-load devices reduced early fall-related events. Mobility aid choice should be individualized and integrated into postoperative rehabilitation planning.

## Supplementary Information

Below is the link to the electronic supplementary material.

Supplementary Material 1

## Data Availability

The datasets used and analyzed during the current study are available from the corresponding author on reasonable request.

## Abbreviations

AATR: Acute Achilles tendon rupture
ERAS: Enhanced recovery after surgery
CAM: Controlled ankle motion
ED: Emergency department
A: U ratio: The affected-to-unaffected calf circumference ratio
PROMs: Patient-reported outcome measures
SLHR 50%: Single-leg heel raise to 50% of the contralateral side
VAS: Visual analog scale
ATRS: Achilles tendon total rupture score
AOFAS: American orthopedic foot and ankle society
MRI: Magnetic resonance imaging
ATRA: Achilles tendon resting angle
BMI: Body mass index
GDRS: Gap distance of the rupture site
DRSTI: Distance from the rupture site to the Achilles tendon insertion
RTW: Return to work
RTLE: Return to light exercise
RTPIE: Return to pre-injury exercise

## References

[1] Ganestam A, Kallemose T, Troelsen A, Barfod KW. Increasing incidence of acute Achilles tendon rupture and a noticeable decline in surgical treatment from 1994 to 2013. A nationwide registry study of 33,160 patients. Knee Surg Sports Traumatol Arthrosc. 2016;24(12):3730–7. 10.1007/s00167-015-3544-5. 25697284 10.1007/s00167-015-3544-5

[2] Bergamin F, Civera M, Rodriguez Reinoso M, Burgio V, Ruiz OG, Surace C. Worldwide incidence and surgical costs of tendon injuries: a systematic review and meta-analysis. Muscle Ligaments Tendons J. 2023;13(1):31. 10.32098/mltj.01.2023.05

[3] Egger AC, Berkowitz MJ. Achilles tendon injuries. Curr Rev Musculoskelet Med. 2017;10(1):72–80. 10.1007/s12178-017-9386-7. 28194638 10.1007/s12178-017-9386-7 PMC5344857

[4] Saxena T, Saxena A, Royds M, Maffulli N. Acute Achilles tendon rupture and chronic tendinopathy surgery: Same tendon, with sex and ethnicity differences. Foot Ankle Surg. 2026;32(2):183–6. 10.1016/j.fas.2025.08.007. 40935779 10.1016/j.fas.2025.08.007

[5] Saxena A, Giai Via A, Grävare Silbernagel K, et al. Current consensus for rehabilitation protocols of the surgically repaired acute mid-substance achilles rupture: a systematic review and recommendations from the GAIT study group. J Foot Ankle Surg. 2022;61(4):855–61. 10.1053/j.jfas.2021.12.008. 35120805 10.1053/j.jfas.2021.12.008

[6] Maffulli N, Irwin AS, Kenward MG, Smith F, Porter RW. Achilles tendon rupture and sciatica: a possible correlation. Br J Sports Med. 1998;32(2):174–7. 10.1136/bjsm.32.2.174. 9631229 10.1136/bjsm.32.2.174 PMC1756077

[7] Maffulli N. Current concepts in the management of subcutaneous tears of the Achilles tendon. Bull Hosp Jt Dis. 1998;57(3):152–8. 9809181

[8] Guevara-Chávez FM, Caballero-Alvarado J, Zavaleta-Corvera C. Efficacy of surgical management versus conservative management to decr ease the incidence of re-rupture in adult patients with achilles tendo n rupture: a systematic review and meta-analysis. Muscles Ligaments Tendons J. 2024;14(1):54–66. 10.32098/mltj.01.2024.06

[9] Maffulli N, Thorpe AP, Smith EW. Magnetic resonance imaging after operative repair of achilles tendon rupture. Scand J Med Sci Sports. 2001;11(3):156–62. 11374429

[10] Maffulli N, Dymond NP, Regine R. Surgical repair of ruptured Achilles tendon in sportsmen and sedentary patients: a longitudinal ultrasound assessment. Int J Sports Med. 1990;11(1):78–84. 10.1055/s-2007-1024767. 2180833 10.1055/s-2007-1024767

[11] Xu XY, Gao S, Lv Y, et al. Duration of immobilisation after Achilles tendon rupture repair by open surgery: a retrospective cohort study. J Orthop Surg Res. 2021;16(1):196. 10.1186/s13018-021-02342-4. 33731160 10.1186/s13018-021-02342-4 PMC7968267

[12] Iborra A, Villanueva M, Ferro D, Palacios J, Maffulli N. Percutaneous ultrasound-guided repair of Achilles tendon rupture: a cadaveric study and preliminary clinical report. J Orthop Surg Res. 2025;20(1):825. 10.1186/s13018-025-06098-z. 40993791 10.1186/s13018-025-06098-z PMC12459053

[13] Maffulli N, Christidis G, Gougoulias N, et al. Percutaneous repair of the Achilles tendon with one knot offers equivalent results as the same procedure with two knots. A comparative prospective study. Br Med Bull. 2025;153(1):ldae019. 10.1093/bmb/ldae019. 10.1093/bmb/ldae019 39673184

[14] Yan XJ, Zhang WH. Enhanced recovery after surgery protocols for minimally invasive treatment of Achilles tendon rupture: Prospective single-center randomized study. World J Orthop. 2024;15(12):1191–9. 10.5312/wjo.v15.i12.1191. 39744729 10.5312/wjo.v15.i12.1191 PMC11686527

[15] Ljungqvist O, Scott M, Fearon KC. Enhanced recovery after surgery: a review. JAMA Surg. 2017;152(3):292–8. 10.1001/jamasurg.2016.4952. 28097305 10.1001/jamasurg.2016.4952

[16] Kang Y, Liu J, Chen H, et al. Enhanced recovery after surgery (ERAS) in elective intertrochanteric fracture patients result in reduced length of hospital stay (LOS) without compromising functional outcome. J Orthop Surg Res. 2019;14(1):209. 10.1186/s13018-019-1238-2. 31288824 10.1186/s13018-019-1238-2 PMC6617739

[17] Gajhede-Knudsen M, Ekstrand J, Magnusson H, Maffulli N. Recurrence of Achilles tendon injuries in elite male football players is more common after early return to play: an 11-year follow-up of the UEFA Champions League injury study. Br J Sports Med. 2013;47(12):763–8. 10.1136/bjsports-2013-092271. 23770660 10.1136/bjsports-2013-092271

[18] Wang R, Huang L, Jiang S, et al. Immediate mobilization after repair of Achilles tendon rupture may increase the incidence of re-rupture: a systematic review and meta-analysis of randomized controlled trials. Int J Surg. 2024;110(6):3888–99. 10.1097/js9.0000000000001305. 38477123 10.1097/JS9.0000000000001305 PMC11175757

[19] Zhao J, Guo W, Zeng X, Kan S. [Research progress of early postoperative rehabilitation for acute Achilles tendon rupture after surgical repair]. Zhongguo Xiu Fu Chong Jian Wai Ke Za Zhi. 2019;33(3):382–6. 10.7507/1002-1892.201807146. 30874399 10.7507/1002-1892.201807146 PMC8337926

[20] Larsson E, Helander KN, Falkheden Henning L, et al. Achilles tendon resting angle is able to detect deficits after an Achilles tendon rupture, but it is not a surrogate for direct measurements of tendon elongation, function or symptoms. Knee Surg Sports Traumatol Arthrosc. 2022;30(12):4250–7. 10.1007/s00167-022-07142-9. 36087127 10.1007/s00167-022-07142-9 PMC9463053

[21] Carmont MR, Grävare Silbernagel K, Brorsson A, Olsson N, Maffulli N, Karlsson J. The Achilles tendon resting angle as an indirect measure of Achilles tendon length following rupture, repair, and rehabilitation. Asia Pac J Sports Med Arthrosc Rehabil Technol. 2015;2(2):49–55. 10.1016/j.asmart.2014.12.002. 29264240 10.1016/j.asmart.2014.12.002 PMC5730640

[22] Cao Y, Li X, Cui Z, Lv Y, Si G. Open surgery and minimally invasive repair of acute Achilles tendon rupture: stratified outcomes based on immobilization duration in a prospective cohort study. J Orthop Surg Res. 2025;20(1):647. 10.1186/s13018-025-06019-0. 40652259 10.1186/s13018-025-06019-0 PMC12255024

[23] Cao Y, Gao S, Cui Z, et al. Comparison of different immobilisation durations following open surgery for acute achilles tendon rupture: a prospective cohort study. J Orthop Surg Res. 2024;19(1):497. 10.1186/s13018-024-04970-y. 39169350 10.1186/s13018-024-04970-y PMC11337624

[24] Cofano E, Colace S, Piro F, et al. Successful functional outcomes and return to sport rate can be achieved after surgery for acute Achilles tendon rupture: A systematic review. J Exp Orthop. 2025;12(4):e70469. 10.1002/jeo2.70469. 41164319 10.1002/jeo2.70469 PMC12560252

[25] Yurdakul G, Atahan MO, Askin A, et al. Intraoperative application of hyaluronic acid in achilles tendon repair: a retrospective cohort study on short-term functional outcomes. Med (Kaunas). 2025;61(10):1816. 10.3390/medicina61101816. 10.3390/medicina61101816 PMC12565747 41155803

[26] Costa ML, Achten J, Marian IR, et al. Plaster cast versus functional brace for non-surgical treatment of Achilles tendon rupture (UKSTAR): a multicentre randomised controlled trial and economic evaluation. Lancet. 2020;395(10222):441–8. 10.1016/s0140-6736(19)32942-3. 32035553 10.1016/S0140-6736(19)32942-3 PMC7016510

[27] Marrone W, Andrews R, Reynolds A, Vignona P, Patel S, O’Malley M. Rehabilitation and return to sports after achilles tendon repair. Int J Sports Phys Ther. 2024;19(9):1152–65. 10.26603/001c.122643. 39246413 10.26603/001c.122643 PMC11379499

[28] Brorsson A, Willy RW, Tranberg R, Grävare Silbernagel K. Heel-rise height deficit 1 year after achilles tendon rupture relates to changes in ankle biomechanics 6 years after injury. Am J Sports Med. 2017;45(13):3060–8. 10.1177/0363546517717698. 28783473 10.1177/0363546517717698

[29] Averkamp BJ, Rees AB, Kalbac T, et al. Comparison of postoperative complications by surgical technique after acute midsubstance achilles tendon repair. Am J Sports Med. 2025;53(12):2898–905. 10.1177/03635465251365520. 40901709 10.1177/03635465251365520

[30] Ochen Y, Beks RB, van Heijl M, et al. Operative treatment versus nonoperative treatment of Achilles tendon ruptures: systematic review and meta-analysis. BMJ. 2019;364:k5120. 10.1136/bmj.k5120. 30617123 10.1136/bmj.k5120 PMC6322065

[31] Maffulli N, Gougoulias N, Maffulli GD, Oliva F, Migliorini F. Slowed-Down Rehabilitation Following Percutaneous Repair of Achilles Tendon Rupture. Foot Ankle Int. 2022;43(2):244–52. 10.1177/10711007211038594. 34581220 10.1177/10711007211038594 PMC8841642

[32] Sharma T, Farrugia P. Early versus late weight bearing & ankle mobilization in the postoperative management of ankle fractures: A systematic review and meta-analysis of randomized controlled trials. Foot Ankle Surg. 2022;28(7):827–35. 10.1016/j.fas.2022.03.003. 35337752 10.1016/j.fas.2022.03.003

[33] Maffulli N, Tallon C, Wong J, Peng Lim K, Bleakney R. No adverse effect of early weight bearing following open repair of acute tears of the Achilles tendon. J Sports Med Phys Fit. 2003;43(3):367–79. 14625519

[34] Heikkinen J, Lantto I, Piilonen J, et al. Tendon length, calf muscle atrophy, and strength deficit after acute achilles tendon rupture: long-term follow-up of patients in a previous study. J Bone Joint Surg Am. 2017;99(18):1509–15. 10.2106/jbjs.16.01491. 28926379 10.2106/JBJS.16.01491

[35] Zhang Y, Tao C, Wang H, Fan Y. Biomechanical effects of human-mobility aid interaction: A narrative review. Gait Posture. 2025;118:1–12. 10.1016/j.gaitpost.2025.01.009. 39842226 10.1016/j.gaitpost.2025.01.009

[36] Rezaei A, Bhat SG, Cheng CH, Pignolo RJ, Lu L, Kaufman KR. Age-related changes in gait, balance, and strength parameters: A cross-sectional study. PLoS ONE. 2024;19(10):e0310764. 10.1371/journal.pone.0310764. 39441815 10.1371/journal.pone.0310764 PMC11498712

[37] Hoeffner R, Agergaard AS, Funaro A, et al. The influence of an orthopaedic walker boot on forefoot force. Foot (Edinb). 2021;46:101739. 10.1016/j.foot.2020.101739. 33285492 10.1016/j.foot.2020.101739

[38] Hullfish TJ, Woods MM, Kwon MP, Boakye LAT, Humbyrd CJ, Baxter JR. The Difference in achilles tendon loading within immobilizing boots based on ankle angle, boot type, and walking speed. Orthop J Sports Med. 2024;12(10):23259671241283806. 10.1177/23259671241283806. 39492878 10.1177/23259671241283806 PMC11529382

[39] Godoy-Santos AL, Bruschini H, Cury J, et al. Fluoroquinolones and the risk of achilles tendon disorders: update on a neglected complication. Urology. 2018;113:20–5. 10.1016/j.urology.2017.10.017. 29074337 10.1016/j.urology.2017.10.017

[40] Maffulli N, Tallon C, Wong J, Lim KP, Bleakney R. Early weightbearing and ankle mobilization after open repair of acute midsubstance tears of the achilles tendon. Am J Sports Med. 2003;31(5):692–700. 10.1177/03635465030310051001. 12975188 10.1177/03635465030310051001

[41] Mashimo S, Nozaki T, Amaha K, et al. Quantitative assessment of calf muscle volume, strength, and quality after achilles tendon rupture repair: a 1-year prospective follow-up study. Am J Sports Med. 2023;51(14):3781–9. 10.1177/03635465231206391. 37960840 10.1177/03635465231206391

[42] Pastori D, Cormaci VM, Marucci S, et al. A Comprehensive Review of Risk Factors for Venous Thromboembolism: From Epidemiology to Pathophysiology. Int J Mol Sci. 2023;24(4):3169. 10.3390/ijms24043169. 10.3390/ijms24043169 PMC9964264 36834580

[43] Aufwerber S, Svedman S, Silbernagel KG, Ackermann PW. Long-term patient outcome is affected by deep venous thrombosis after Achilles tendon rupture repair. Knee Surg Sports Traumatol Arthrosc. 2024;32(8):2184–93. 10.1002/ksa.12240. 38796725 10.1002/ksa.12240

[44] Saxena A, Ewen B, Maffulli N. Rehabilitation of the operated achilles tendon: parameters for predicting return to activity. J Foot Ankle Surg. 2011;50(1):37–40. 10.1053/j.jfas.2010.10.008. 21106412 10.1053/j.jfas.2010.10.008

[45] Jaeschke R, Singer J, Guyatt GH. Measurement of health status. Ascertaining the minimal clinically important difference. Control Clin Trials. 1989;10(4):407–15. 10.1016/0197-2456(89)90005-6. 2691207 10.1016/0197-2456(89)90005-6

[46] Farrar JT, Young JP Jr., LaMoreaux L, Werth JL, Poole MR. Clinical importance of changes in chronic pain intensity measured on an 11-point numerical pain rating scale. Pain. 2001;94(2):149–58. 10.1016/s0304-3959(01)00349-9. 11690728 10.1016/S0304-3959(01)00349-9

[47] Chen C, Li Z, Zhang Y, et al. What’s the clinical significance of VAS, AOFAS, and SF-36 in progressive collapsing foot deformity. Foot Ankle Surg. 2024;30(2):103–9. 10.1016/j.fas.2023.10.002. 37858492 10.1016/j.fas.2023.10.002

[48] Westin O, Svensson M, Nilsson Helander K, et al. Cost-effectiveness analysis of surgical versus non-surgical management of acute Achilles tendon ruptures. Knee Surg Sports Traumatol Arthrosc. 2018;26(10):3074–82. 10.1007/s00167-018-4953-z. 29696317 10.1007/s00167-018-4953-z PMC6154020

[49] Manocha RHK, MacGillivray MK, Eshraghi M, Sawatzky BJ. Injuries associated with crutch use: a narrative review. PM & R. 2021;13(10):1176–92. 10.1002/pmrj.12514. 33094912 10.1002/pmrj.12514

