# Supplementary material for: Comparative analysis of different mobility aids in the early accelerated rehabilitation phase following acute achilles tendon repair surgery: a prospective cohort study
Source: J Orthop Surg Res. 2026 Apr 27;21:279. doi: 10.1186/s13018-026-06881-6 (PMC13123103; doi:10.1186/s13018-026-06881-6)
Supplement: Supplementary file 1 — Supplementary Material 1 [file 13018_2026_6881_MOESM1_ESM.docx]

Supplementary Table S1: **Accelerated rehabilitation protocol**

| **Postoperative period** | **Rehabilitation Training Plan** |
| --- | --- |
| 0-2 weeks after surgery | Immobilization with a brace, weight-bearing and activity are strictly prohibited. |
| Immediately after removing the brace | Ankle mobilization, wearing a CAM boot, and commencing prescribed mobility aids |
| 2-4 weeks after surgery | Standing up for 1 hour per day |
| 4-6 weeks after surgery | Standing up for 2 hours per day,  Deep squat |
| Approximately 6 weeks after surgery | Transition to Axillary Crutches* |
| 6-8 weeks after surgery | Double-legged heel raises  Walking less than 1000 steps on flat ground |
| 8-12 weeks after surgery | Single-legged heel raises  Walking less than 2000 steps on flat ground |
| Approximately 12 weeks after surgery | Weaning off mobility aids |
| 2 weeks after successfully performing single-legged heel raises | Jogging |
| 4 weeks after successfully performing jogging | More vigorous training |

*After week 6, all patients were transitioned to axillary crutches regardless of the initial mobility aid used during weeks 3–6. CAM, controlled ankle motion.

Supplementary Table S2: **Stratified analysis of unplanned ED visits by age group during postoperative weeks 3–6**

| **Age group** | **Mobility aids** | **ED visits, n** | **Participants, n** | **Rates** | **P value** |
| --- | --- | --- | --- | --- | --- |
| < 50 years | Low-load aids | 3 | 75 | 4.0% |  |
|  | High-load aids | 16 | 102 | 15.7% | 0.015 |
|  | Total | 19 | 177 | 10.7% |  |
| ≥ 50 years | Low-load aids | 1 | 9 | 11.1% |  |
|  | High-load aids | 5 | 12 | 41.7% | 0.153 |
|  | Total | 6 | 21 | 28.6% | 0.025 |

A further exploratory stratified analysis of unplanned ED visits during postoperative weeks 3–6 showed event rates ascending from 4.0% (<50 years, low-load aids) to 11.1% (≥50 years, low-load aids), 15.7% (<50 years, high-load aids), and 41.7% (≥50 years, high-load aids). Among patients aged <50 years, high-load aids were associated with a significantly higher ED visit rate (15.7% vs 4.0%, p = 0.015); a similar numerical trend was observed in those aged ≥50 years (41.7% vs 11.1%, p = 0.153). When both mobility-aid categories were combined, patients aged ≥50 years had a higher overall ED visit rate than those aged <50 years (28.6% vs 10.7%, p = 0.025).

Data are presented as n/N (%). Low-load aids included wheelchairs and knee scooters. High-load aids included axillary crutches and the leg support walker. Given the limited sample size among older participants, these findings should be considered exploratory and interpreted with caution.

Supplementary Table S3: **Adjusted multivariable logistic regression analysis for unplanned ED visits during postoperative weeks 3–6.**

| **Variables** | **B** | **SE** | **Wald** | **df** | **Adjusted OR (95% CI)** | **P value** |
| --- | --- | --- | --- | --- | --- | --- |
| Mobility Aids |  |  | 7.964 | 3 |  | 0.046 |
| *Knee Scooter* | 1.308 | 1.348 | 0.941 | 1 | 3.697 (0.263–51.939) | 0.332 |
| *Axillary Crutches* | 2.545 | 1.103 | 5.327 | 1 | 12.746 (1.468–110.683) | 0.021 |
| *LS Walker* | 2.155 | 1.022 | 4.445 | 1 | 8.628 (1.164–63.968) | 0.035 |
| Age | 2.399 | 0.689 | 12.118 | 1 | 11.013 (2.856–42.467) | < 0.001 |
| Sex | -0.150 | 0.082 | 3.221 | 1 | 0.861 (0.733–1.011) | 0.068 |
| BMI | -0.011 | 0.106 | 0.011 | 1 | 0.989 (0.805–1.215) | 0.917 |
| Activity level | 0.070 | 0.524 | 0.018 | 1 | 1.073 (0.303–3.799) | 0.893 |
| Diabetes | 1.016 | 0.606 | 2.805 | 1 | 2.761 (0.841–9.063) | 0.094 |
| Constant | -2.847 | 3.091 | 0.848 | 1 | 0.058 | 0.357 |

A further adjusted multivariable logistic regression analysis of unplanned ED visits during postoperative weeks 3–6 showed that mobility aid type was significantly associated with the outcome overall (P = 0.046). Using wheelchair as the reference, axillary crutches (adjusted OR = 12.746, 95% CI 1.468–110.683, p = 0.021) and LS walker (adjusted OR = 8.628, 95% CI 1.164–63.968, p = 0.035) were associated with significantly higher odds, whereas knee scooter was not (adjusted OR = 3.697, 95% CI 0.263–51.939, p = 0.332). Age ≥50 years was independently associated with a higher likelihood of unplanned ED visits (adjusted OR = 11.013, 95% CI 2.856–42.467, p < 0.001). No significant associations were observed for sex, BMI, activity level, or diabetes (all p > 0.05).

Data are presented as regression coefficient (B), standard error (SE), Wald statistic, adjusted odds ratio (OR), and 95% confidence interval (CI). The model was adjusted for mobility aid type, age group (<50 vs ≥50 years), sex (female vs male), BMI, activity level (<3 vs ≥3 sessions/week), and diabetes (no vs yes). Wheelchair was used as the reference category for mobility aid type.
